# Diode laser gingival peeling. case report

**DOI:** 10.21142/2523-2754-1402-2026-292

**Published:** 2026-04-04

**Authors:** Daniel Enciso Lupuche, Emily Rojas Trebejo, Olinda Huapaya Paricoto, María Eugenia Guerrero Acevedo

**Affiliations:** 1 Faculty of Dentistry, Universidad Nacional Mayor de San Marcos. Lima, Peru. daniel.enciso@unmsm.edu.pe, emily.rojas2@unmsm.edu.pe Universidad Nacional Mayor de San Marcos Faculty of Dentistry Universidad Nacional Mayor de San Marcos Lima Peru daniel.enciso@unmsm.edu.pe emily.rojas2@unmsm.edu.pe; 2 OMEQ Research Group, Faculty of Dentistry, Universidad Nacional Mayor de San Marcos. Lima, Peru. dhuapayap@unmsm.edu.pe, mguerreroac@unmsm.edu.pe Universidad Nacional Mayor de San Marcos OMEQ Research Group Faculty of Dentistry Universidad Nacional Mayor de San Marcos Lima Peru dhuapayap@unmsm.edu.pe mguerreroac@unmsm.edu.pe

**Keywords:** diode laser, peeling, gingiva, melanosis, láser diodo, *peeling*, melanosis

## Abstract

In modern dentistry, esthetics plays a crucial role in the well-being of patients, since a harmonious smile, including lips, teeth and gums, directly impacts self-confidence. The gingiva normally has a pink or coral color but can darken due to endogenous factors (such as genetics and melanogenesis) or exogenous factors (such as medications, smoking and systemic diseases). When the gingiva darkens excessively, this is gingival hyperpigmentation, which is characterized by brown spots. To correct this, several treatments have been developed, including laser surgery, which is effective, precise and less invasive. In this context, a case is presented in which a diode laser was used to perform a “gingival peel” at a public university in Peru. The case is presented of a 64-year-old patient of African descent, smoker and native of Lima, with a history of Poliomyelitis and a surgical intervention on the spine due to the sequelae, which caused motor disability. The patient went to the Comprehensive Adult Clinic IV (CIA IV) of the Faculty of Dentistry of the Universidad Nacional Mayor de San Marcos (UNMSM), accompanied by her friend, due to the absence of teeth and the lack of esthetics in the anterior sector. The effectiveness of laser therapy in the treatment of gingival peeling is concluded, highlighting the key role of the diode laser in obtaining positive results. The procedure was safe, simple and fast, according to several sources. In addition, it favored a postoperative period without pain or bleeding, reduced healing time and allowed recovery with minimal discomfort, improving patient satisfaction and comfort.

## RESUMEN

En la odontología moderna, la estética juega un papel crucial en el bienestar de los pacientes, ya que una sonrisa armoniosa, que incluye labios, dientes y encías, impacta directamente en la autoconfianza. La encía normalmente tiene un color rosado o coral, pero puede oscurecerse debido a factores endógenos (como la genética y la melanogénesis) o exógenos (como medicamentos, tabaquismo y enfermedades sistémicas). Cuando la encía se oscurece excesivamente, se trata de hiperpigmentación gingival, que se caracteriza por manchas de color marrón. Para corregir esto, se han desarrollado varios tratamientos, incluyendo la cirugía láser, que es eficaz, precisa y menos invasiva. En este contexto, se presenta un caso en el que se utilizó un láser de diodo para realizar un *peeling* gingival en una universidad pública del Perú. Se presenta el caso de una paciente de 64 años, afrodescendiente, fumadora y natural de Lima, con antecedentes de poliomielitis y una intervención quirúrgica en la columna debido a las secuelas, lo que le ocasiona discapacidad motora. La paciente acudió a la Clínica Integral del Adulto IV (CIA IV) de la Facultad de Odontología de la Universidad Nacional Mayor de San Marcos (UNMSM), acompañada de su amiga, debido a la ausencia de dientes y la falta de estética en el sector anterior. Se concluye la efectividad de la terapia con láser en el tratamiento de *peeling* gingival, y destaca el papel clave del láser de diodo para obtener resultados positivos. El procedimiento fue seguro, sencillo y rápido, según diversas fuentes. Además, favoreció un posoperatorio sin dolor ni sangrado, redujo el tiempo de cicatrización y permitió una recuperación con mínimas molestias, mejorando la satisfacción y el confort del paciente.

## INTRODUCTION

Currently, aesthetics in dentistry has become important for patients' well-being in addition to their oral health, since a smile is one of the factors that psychologically and aesthetically influences self-confidence. Besides being composed of lips and teeth, the gingival tissues also contribute to its composition, creating aesthetic harmony in both shape and color. Under normal conditions, the gingiva presents a pale pink or coral color with a stippled texture known as "orange peel," although it can also exhibit other color variations, such as pale bluish-purple ^1^^,^^2^.

The discoloration or darkening that commonly occurs is influenced by various endogenous and exogenous factors. Endogenous factors include cellular activity such as the degree of vascularization, keratinization of the stratum corneum, thickness of the gingival epithelium, melanogenesis and its distribution, and racial type, which is more common in people of African, Asian, or Mediterranean descent. Exogenous factors are associated with the use of medications such as tetracyclines, antimalarials, ketoconazole, and zidovudine; heavy smoking, also known as "smoker's melanosis"; and systemic diseases such as Addison's disease, neurofibromatosis, and Peutz-Jeghers syndrome. If the gums appear darker than normal or "black," this indicates gingival hyperpigmentation caused by melanosis, which is an excessive release of melanin pigments in the gingival epithelium. Clinically, it appears as asymptomatic, single or multiple, well-defined macules that tend to be dark or light brown in color and vary in size, located particularly on the gingiva. This discoloration affects people aesthetically, so different treatments have been developed to depigment the tissue, such as diamond bur abrasion, cryosurgery, chemical treatment, electrosurgery, and laser surgery ^1^^,^^3^.

Laser surgery, also known as "gingival peeling," has been used in dental procedures in recent years. The laser acts on melanocytes, the cells responsible for producing melanin, which absorb light energy and transform it into heat through photothermolysis. Its use has been highlighted for being minimally invasive, precise, fast, causing little bleeding, and less painful. Lasers such as diode, carbon diode, and neodymium- YAG (yttrium aluminum garnet) lasers are used for this purpose ^4^.

This case report aims to highlight a gingival peeling procedure using a diode laser, performed for the first time at a public university in Peru.

## CASE REPORT

We present the case of a 64-year-old Afro-Peruvian female patient, a smoker, originally from Lima, with a history of poliomyelitis. Due to the resulting complications, she underwent spinal surgery and currently has a motor disability. She visited the Comprehensive Adult Clinic IV (CIA IV) of the Faculty of Dentistry at the National University of San Marcos (UNMSM), accompanied by a friend. The consultation was prompted by missing teeth and concerns about the aesthetics of her anterior teeth.

### Clinical Examination

**Figure 1:**
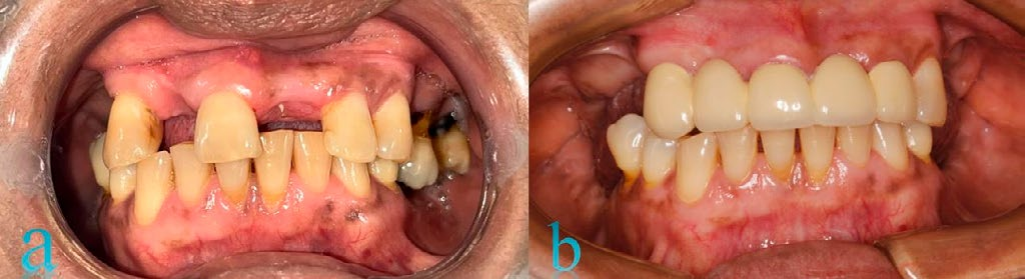
Intraoral clinical examination a) Frontal view before prosthetic treatment; b) Frontal view after prosthetic treatment.

During the intraoral examination, regular oral hygiene was observed, along with the presence of gingival melanosis in both left arches. The patient presented with edentulous spaces and some teeth with poor prognosis (Figure 1). Therefore, a comprehensive treatment plan was prescribed, beginning with biofilm control through proper brushing techniques. Following this, root canal treatment was performed on one of the abutment teeth to prepare for prosthetic treatment. Once completed, the patient was advised to undergo gingival peeling to eliminate the melanosis present in both arches.

### Surgical Procedure

The surgical procedure was scheduled in the undergraduate operating room of the Faculty of Dentistry at UNMSM, after obtaining the patient's informed consent. Rigorous antiseptic and aseptic measures were adopted in the preparation of the surgical site.

The procedure began with programming the 976 nm diode laser (LX16 Plus Woodpecker®) at a frequency of 10 Hz and a valid power of 1.00 W. The patient did not require pre-treatment and rinsed with 0.12% chlorhexidine for 1 minute prior to the procedure without the application of local anesthesia, following the protocols of most of the literature.

**Figure 2:**
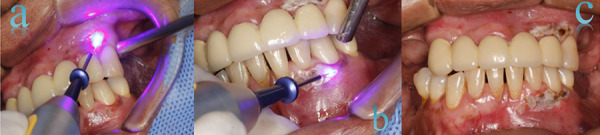
Application of the diode laser. a) Maxillary sector; b) Mandibular sector

The surgical procedure consisted of using the laser tip to gently scrape the center of the lesion. Since the objective was to remove the melanotic lesion without affecting the bone tissue, care was taken not to keep the laser in the same place for too long or to penetrate too deeply. (Figure 2) However, ten minutes after the laser application, the patient began to experience pain, so she was given 2% lidocaine infiltration anesthesia without epinephrine in the anterior alveolar nerves to prevent vasoconstriction of the tissues.

Follow-up visits were conducted 3 and 7 days after the procedure, showing adequate healing (Figures 4 and 5). At 12 months (Figure 6), there was no recurrence and no evidence of gingival melanosis.

**Figure 3:**
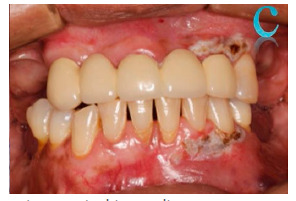
Postoperative surgical immediate

**Figure 4:**
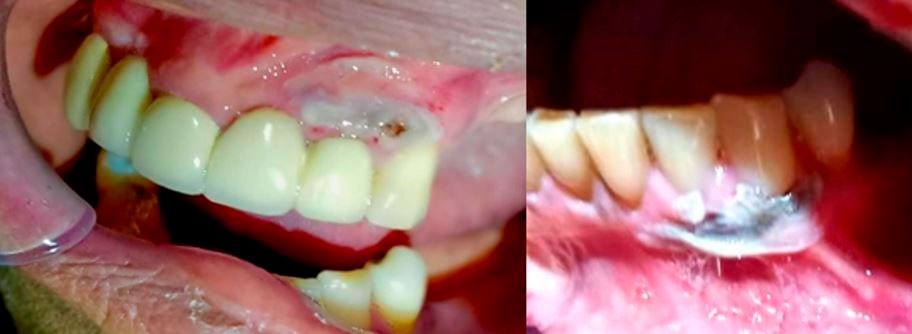
3-day follow-up. a) Maxillary sector. b) Mandibular sector

**Figure 5:**
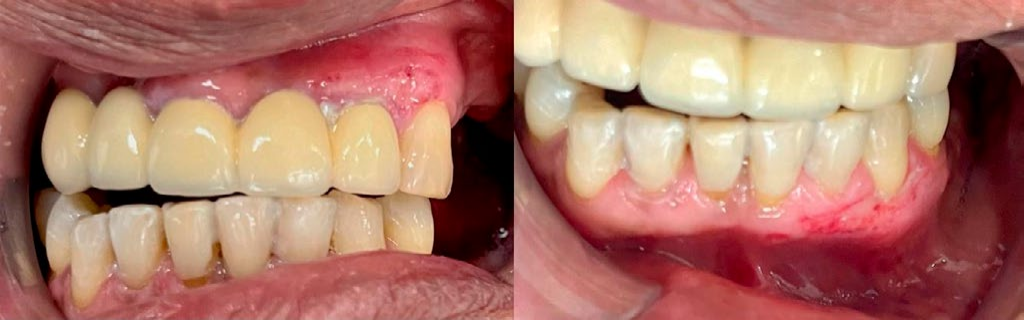
7-day follow-up. a) Maxillary sector. b) Mandibular sector

**Figure 6:**
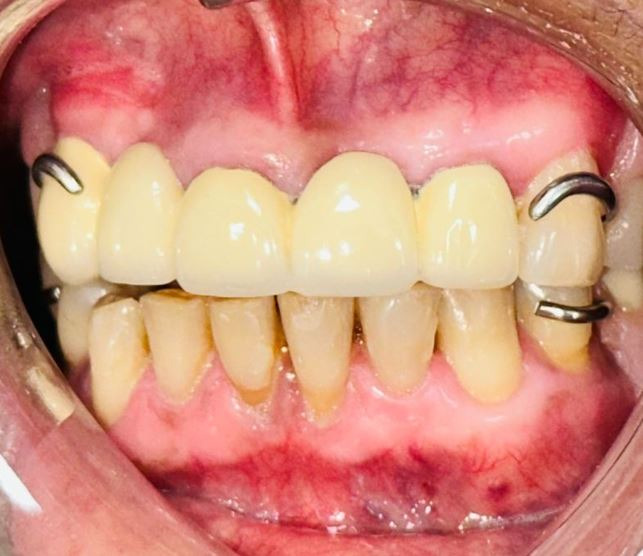
1-year follow-up. Note the absence of relapses and no evidence of gingival melanosis.

## DISCUSSION

Gingival melanosis is a common benign condition in clinical practice, with a prevalence ranging from 0% to 89%, depending on the population studied and ethnic, genetic, and environmental factors ^5^^-^^7^. Although it is not a disease per se, its aesthetic impact can cause discomfort and affect the patient's self-confidence, especially in cases of high gingival smile or excessive exposure of the gingival tissue ^8^.

The treatment of gingival hyperpigmentation aims to restore the aesthetic harmony of the dentogingival complex. Over the last few decades, different therapeutic modalities have been described: mechanical techniques (diamond bur abrasion), chemical techniques (phenol-alcohol), surgical techniques (scalpel), cryosurgery, and, more recently, the use of lasers ^3^^,^^4^. Among these, the diode laser has proven to be a safe, predictable, and minimally invasive option, capable of producing precise removal of the pigmented epithelial layer through selective photothermolysis of melanocytes ^10^^,^^11^.

The mechanism of action of the diode laser consists of converting the light energy absorbed by tissue chromophores into heat, which causes the controlled destruction of melanocytes without damaging underlying structures. This principle allows for homogeneous ablation, a clean surgical field, and excellent hemostasis, as well as a more comfortable postoperative period. Compared to conventional scalpel surgery, the laser technique generates less bleeding, edema, and pain, while also reducing the risk of infection and accelerating healing through biological stimulation of fibroblasts ^3^^,^^10^^,^^13^.

The case presented clearly illustrates the clinical advantages of diode laser in a real-world setting. The patient, with a history of poliomyelitis and motor disability, presented a clinical challenge due to both her physical limitations and the need for a brief, clean, and bloodless procedure. In this regard, laser technology allowed the treatment to be performed under optimal conditions, minimizing intraoperative discomfort and facilitating a rapid recovery. This demonstrates that laser treatment has not only aesthetic value but also functional and therapeutic benefits, especially in systemically compromised patients or those with reduced mobility, for whom traditional methods might involve greater risk or difficulty.

Furthermore, the application of this procedure in an academic setting such as the Faculty of Dentistry at the National University of San Marcos demonstrates that integrating lasers into university education is both feasible and valuable. Access to advanced technology allows students and faculty to acquire skills in modern therapies, expanding the range of conservative and aesthetic treatments within the public healthcare system. This is particularly relevant in the Peruvian and Latin American context, where minimally invasive dentistry continues to grow.

In recent literature, Inchingolo et al. (2024) compared diode lasers with surgical scalpels and found similar clinical results in gingival depigmentation, but with better bleeding control and less pain in the laser-treated group ^10^. Similarly, Bing et al. (2024) and Mojahedi et al. (2023) demonstrated that diode lasers, at different wavelengths (808 nm, 445 nm, and 980 nm), offer predictable results, with minimal carbonization and controlled thermal damage ^11^^,^^12^. In the present case, the 976 nm laser showed an immediate superficial carbonization effect without subsequent complications, confirming the safety of the clinical protocol used.

Postoperative monitoring showed a favorable outcome, characterized by the absence of pain, bleeding, or edema, and adequate epithelial healing. These results are consistent with the findings of Altayeb et al. (2021), who observed faster and less traumatic recovery with diode laser compared to other wavelengths ^14^. Furthermore, no signs of repigmentation were detected at 12-month follow-up, which reinforces the findings reported by Haque et al. (2024) and Hassan et al. (2022), who documented significantly lower recurrence rates with laser use ^19^^,^^20^.

Repigmentation is one of the main challenges in gingival depigmentation, as it depends on factors such as the technique, the type of laser, the depth of ablation, the patient's race and habits (e.g., smoking). In this case, the stability of the results can be attributed to the conservative protocol applied, adequate postoperative control, and the use of safe parameters (1 W, 976 nm), which is consistent with the guidelines proposed by Zahid (2025) and other authors ^13^.

From a clinical perspective, the diode laser offers tangible advantages over conventional methods: it reduces the need for anesthesia, improves operative visibility, decreases the risk of cross-infection, and allows for precise thermal control. Furthermore, it represents a versatile tool in periodontics, oral surgery, and gingival aesthetics, with benefits for both the practitioner and the patient ^3^^,^^10^^,^^13^^,^^18^.

## CONCLUSION

This clinical case demonstrates the efficacy and safety of diode laser as a therapeutic tool for the treatment of gingival melanosis. The procedure was minimally invasive, quick, and with excellent hemostatic control, allowing for painless and bloodless recovery.

In addition to the aesthetic benefits, this experience demonstrates the applicability of lasers in patients with systemic conditions or motor limitations, offering a safe and predictable alternative to conventional techniques. The incorporation of lasers in academic and public clinical settings represents a significant advancement in minimally invasive dentistry, contributing to improved quality of life and aesthetic confidence for patients.
